# Preventive Hoof Trimming and Animal-Based Welfare Measures Influence the Time to First Lameness Event and Hoof Lesion Prevalence in Dairy Cows

**DOI:** 10.3389/fvets.2021.631844

**Published:** 2021-06-11

**Authors:** Mohammed B. Sadiq, Siti Z. Ramanoon, Wan Mastura M. Shaik Mossadeq, Rozaihan Mansor, Sharifah S. Syed-Hussain

**Affiliations:** ^1^Department of Farm and Exotic Animal Medicine and Surgery, Faculty of Veterinary Medicine, Universiti Putra Malaysia, Serdang, Malaysia; ^2^Center of Excellence (Ruminant), Faculty of Veterinary Medicine, Universiti Putra Malaysia, Serdang, Malaysia; ^3^Department of Veterinary Pre-Clinical Science, Faculty of Veterinary Medicine, Universiti Putra Malaysia, Serdang, Malaysia; ^4^Department of Veterinary Clinical Studies, Faculty of Veterinary Medicine, Universiti Putra Malaysia, Serdang, Malaysia

**Keywords:** lameness, hoof lesions, hoof trimming, animal welfare, dairy cows

## Abstract

**Background:** The objectives of this study were to, (1) investigate the impact of the Dutch five-step hoof trimming (HT) technique on time to lameness and hoof lesion prevalence in grazing (GR) and non-grazing (NGR) dairy cows, and (2) determine the association between potential benefits of HT and animal-based welfare measures during lactation. A total of 520 non-lame cows without hoof lesions from 5 dairy farms (GR = 2, NGR = 3) were enrolled at early (within 30 days in milk; DIM) and late lactation (above 200 DIM), and randomly allocated to either trimmed (HGR or HNGR) or control groups (CON-GR and CON-NGR). Locomotion scores, body condition, hock condition, leg hygiene, and hoof health were assessed at monthly intervals until the following 270 days in milk. The data were analyzed using Kaplan-Meier survival analysis, multivariable Cox, and logistic regression models. The overall incidence rate of lameness was 36.2 cases/100 cows/month, with corresponding rates of 27.4, 31.9, 48.4, and 45.8 cases/100 cows/month in HGR, HNGR, CON-GR, and CON-NGR, respectively. Time to first lameness event was significantly higher in HGR (mean ± S.E; 8.12 ± 0.15) compared to CON-GR (7.36 ± 0.26), and in HNGR (8.05 ± 0.16) compared to CON-NGR (7.39 ± 0.23). The prevalence of hoof lesions in the enrolled cows was 36.9%, with a higher occurrence in CON-GR (48.8%) than HGR (23.2%), and in CON-NGR (52.6%) compared to HNGR (32.2%). The majority of hoof lesions were non-infectious in grazing (HGR vs. CON-GR; 21.3 vs. 33.3%) and non-grazing herds (HNGR vs. CON-NGR; 25.0 vs. 40.4%). The risk of lameness was higher in underconditioned cows (Hazard ratio; HR = 3.1, 95% CI 1.2–7.4), presence of hoof lesion (HR = 33.1, 95% CI 17.6–62.5), and there was variation between farms. Aside HT, lower parity (OR = 0.4, 95% CI 0.2–0.8), normal hock condition (OR = 0.06; 95% 0.01–0.29), and absence of overgrown hoof (OR = 0.4; 95% 0.2–0.7) were protective against non-infectious hoof lesions. Functional HT is beneficial as a lameness preventive strategy during lactation; however, ensuring older cows are in good body condition and free from hock injuries are equally important.

## Introduction

Lameness is amongst the 3 most common health issues affecting dairy cows after mastitis and infertility (1, 2). It remains a financial burden to dairy farmers, with significant economic loss attributed to impaired milk yield, low reproduction performance, high culling risk, and treatment costs (3–5). Most lameness cases involve hoof pathologies, although their presence may not elicit detectable changes in cows' gait (4, 6). Thus, lameness may be seen as an indicator of hoof lesion, which is often painful and contributes to the poor welfare of dairy cattle (7, 8).

Animal-based welfare measures (ABWM) are vital parameters in assessing the well-being and performance of dairy cows (9). For instance, individual cow characteristics such as body condition, leg hygiene, hock condition, and lying behavior have been associated with lameness levels on dairies (6, 7, 10). Cow-level factors relating to body condition including combined depth of the digital cushion and corium influenced the development of hoof horn disruptive lesions during lactation (11, 12). However, there is a scarcity of data regarding the variation of these measures with intervention targeted on lameness control. Moreover, ABWM need to be monitored during lactation to identify the variation of welfare outcomes and developing appropriate management plans (13).

Lameness levels vary under different management systems, with studies reporting higher prevalence in non-grazing or confined cows than those kept on pasture-based herds (14, 15). Factors such as prolonged standing time, reduced lying time, and little exercise were suggested to enhance the development of hoof lesions in confined and non-grazing dairy cows (16). These events contribute to detrimental hoof traits including disproportionate heel height, too-long dorsal wall, greater imbalance in weight distribution between the front and hind hooves (3, 17), and increasing the need for preventive or curative hoof trimming (HT) (18).

The five-step Dutch method (i.e., functional trimming) is widely practiced in the dairy industry (19). The HT technique advocates for relatively leveled abaxial and axial walls of the claw, and they are presented perpendicular to the metatarsals (17). Few authors have reported the benefits of functional HT as a lameness control strategy on dairies. Cows that were trimmed at mid-lactation had a lower incidence of lameness (20) and lower odds of hoof lesions (21) compared to control groups. The incidence of hoof horn lesions was significantly lower in farms conducting preventive HT compared to farms lacking such practice (20), while cows trimmed at late lactation had lower odds of developing sole ulcers in the subsequent lactation (21). However, based on the study designs and lack of information on previous lameness events, lesion history, and characteristics of the enrolled cows, attributing better hoof health to preventive HT is limited. Additionally, the benefits of preventive HT as a lameness management strategy in cows managed under confined and grazing conditions have not been widely investigated. By enrolling cows with reliable information on lameness history and monitoring of ABWMs, variation in either lameness or hoof lesion levels can be identified, and targeted welfare management plans can be implemented. Therefore, the objectives of this study were to, (1) investigate the impact of the Dutch five-step HT technique on time to lameness and lesion prevalence in grazing (GR) and non-grazing (NGR) dairy cows, and (2) determine the animal-based factors that may influence the potential benefits of HT during lactation.

## Materials and Methods

### Selection of Farms

This study is part of a large project including several observational studies on the epidemiology and effective preventive measures for lameness control in Malaysian dairy farms. A total of 14 dairy farmers from six states in Peninsular Malaysia were contacted *via* a phone and email directory obtained from the Divisional Department of Veterinary Services (DVS), and they were briefed about the study objectives, inclusion criteria, and methodology. The inclusion criteria entailed farmers' consent to participate, large herd size (>150 cows), adequate farms' health and production records, availability of HT chute (manual or hydraulic system), and either confined housing or provision of pasture access. Eight of the farmers agreed to participate. Upon farm visits, the herds were re-evaluated for compliance with the inclusion criteria, and five farms located in five states in Peninsular Malaysia (Selangor, Negeri Sembilan, Melaka, Johor, and Pahang) were finally enrolled in the study. The data collection took place from October 2018 to December 2019.

### Farm Management Practices

Farm characteristics and management practices (e.g., herd size, number of milking cows, and number of staff, HT services, feeding pattern, cleaning frequency, milking technique, footbath usage, and animal source) were assessed through a structured interview with the farm owner or manager. The interview session was conducted by a single researcher in all the selected farms. Factors related to barn design (e.g., flooring type, floor cleanliness, and slipperiness, distance to milking point, stocking density) were evaluated using a modified farm inspection protocol developed by Grandin (22) and Solano et al. (23) as presented in Supplementary Table 1.

Two of the farms (Farm C and D) were categorized as GR herds based on the provision for external grazing for 3–6 h daily all year round. The other three farms (Farm A, B, and E) practiced the NGR system as cows were completely housed indoor all year round (Table 1). In addition to pasture access, Farm D had an outdoor exercise area with compost bedding. All the farms used fans as a heat-abatement strategy. Also, concrete floors with installed rubber mats were present at the holding, resting, and milking pens in all the enrolled farms. The herd size ranged from 125 to 2,800 cows, while the 305-day milk yield/cow ranged from 3,050 to 5,490 kg. Professional hoof trimmers were invited on a timely basis for the management of lame cows in four farms (Farm A, B, C, and E), and only one farm (Farm D) had on-farm hoof care and trimming unit.

**Table 1:** Herd level factors of the non-grazing (*n* = 3) and grazing (*n* = 2) farms enrolled in the study.

	**Non-grazing**			**Grazing**	
**Factors**	**Farm A**	**Farm B**	**Farm E**	**Farm C**	**Farm D**
Stocking density	0.8 cow/stall	1.0 cow/stall	1.3 cow/stall	0.94 cow/stall	1.2 cow/stall
Milking technique	Milking machine	Milking machine	Milking machine	Milking machine	Milking machine
Cleaning frequency	Twice/day	>Twice/day	Twice/day	Twice/day	Twice/day
Outdoor exercise	No	No	No	No	Yes
Flooring-related factors					
Barn floor type	Concrete/RM	Concrete/RM	Concrete/RM	Concrete/RM	Concrete/RM
Walkway floor type	Concrete/RM	Concrete/RM	Concrete/RM	Concrete/RM	Concrete/RM
Slipperiness	Non-slippery	Non-slippery	Non-slippery	Non-slippery	Non-slippery
Cleanliness	Clean	Dirty	Clean	Clean	Clean
Cleaning method	Vehicular manual	Manual	Manual	Manual	Automated scraper
Average distance from barn to milking point	30 m	45 m	50 m	40–60 m	65–80 m
Source of replacement cows	Australia	Australia	Australia and local	Australia, Thailand, and local	Australia
Hoof trimming practice	Except during treatment	Except during treatment	1/year	Except during treatment	2/year
Footbath	Yes	Yes	Yes	Yes	Yes
305-day milk yield	4,230	3,355	3,050	3,250	5,490
Herd size	125	180	252	350	2,800
Number of milking cows	92	121	154	174	1,210

*RM, rubber mats*.

### Study Design and Sample Size Calculation

This study employed a prospective longitudinal approach including 4 cohort groups; hoof-trimmed from GR farms (HGR), hoof-trimmed from NGR farms (HNGR), non-trimmed/control from GR (CON-GR), non-trimmed/control from NGR (CON-NGR). Hence, each farm had a proportion of trimmed and non-trimmed cows. The cows were enrolled at early (within 30 DIM) or late lactation (≥200 DIM). The follow-up period was 9 months from enrollment, which was sufficient for the cows to be observed during the high-risk period for lameness in the present and subsequent lactation. Also, the period was selected considering the scheduled time for preventive HT in farm B. The required sample size per group was calculated by assuming a precision level of 5%, power of 80%, and expected lameness incidence of 40 and 20% in control groups from NGR and GR farms, respectively. The estimated sample size per group was increased from 110 to 120 animals to adjust for loss to follow-up, by assuming that 10% of the animals will be culled during the study period.

### Cow Selection and Enrollment

The inclusion criteria entailed sound locomotion score (LS < 3), moderate BCS (2.5–4.0) based on the 5-point scoring scales developed by Sprecher et al. (24) and Vasseur et al. (25), respectively, presence of healthy hooves, and indications for maintenance HT (overgrown hoof, unbalanced sole surface, and disproportional heel height). On the first day of visits, all the lactating cows in each farm were assessed for locomotion scores. The cows were observed and assessed for locomotion scores one at a time while they exited the milking parlor. LS was recorded when cows completed a minimum of four steps and undisturbed while walking on a flat and non-slippery floor surface. Cows were considered for selection when presented with sound mobility (LS < 3) and farmers' consent for such animals remaining in the herd until the next lactation. Thereafter, lame cows and those affected with other health issues or had been treated using non-steroidal anti-inflammatory drugs and/or antibiotics 2 weeks before the visit were excluded.

The hoof health of the cows was examined. A multi-purpose HT chute was present in four of the enrolled farms (Farms A, B, C, and D). To enable the cows to get accustomed to the trimming chute, they were allowed to walk through the facility when returning to resting barns after milking. This was conducted twice daily for 3 days before enrollment. In Farm E, cows were examined on a tilting table HT facility (hydraulic system). The cows' limbs were restrained and their hooves were assessed to ensure the absence of lesions before enrollment. The dorsal wall length was measured using a claw check device based on the distance from the proximal aspect of the perioplic horn to the distal end of the dorsal wall. Values ranging from 7 to 9 cm were considered normal, whereas values >9 cm were recorded as overgrown (26).

Other cows' characteristics such as leg hygiene and hock condition were recorded. Leg hygiene was measured using the 3-point scoring scale described by Vasseur et al. (27) where 1 = clean (absence of manure flecks on the lower limbs, upper limbs, and upper flank region), 2 = dirty (distinct splash of manure around the area), and 3 = very dirty (confluent plagues of manure). Hock condition was assessed by scoring (3-point scale) the fore and hind limbs based on the condition of the area around the tarsal (hock) and carpal (knee) joints, where 1 = healthy, 2 = balding or mild swelling, 3 = swollen or open wound (28).

### Hoof Trimming and Animal Placement into Groups

Upon tossing a coin, the selected cows in each farm were randomly allocated into either the trimmed or control group. Cows selected for trimming were restrained in the HT facility and their hooves were trimmed using the five-step Dutch method (28). Briefly, overgrown hooves were identified, marked using a claw check, and reduced to normal length using a hoof nipper. The inner claw (medial claw) of the hindfoot was trimmed before proceeding to the outer claw (lateral claw) and vice versa for the front foot. A HT knife (Kruuse®) was used to pair and level the sole and heel region. For standardization, a little modification of the model described by other authors (29) was applied to pin-point the paring from axial to abaxial aspects of the claw (40 mm away). The cow enrollment and HT in farms A, C, D, and E were completed after 3 visits to each farm by the same veterinarian. In farm B, HT was conducted by the veterinarian and farms' professional hoof trimmers trained on how to apply the trimming technique employed in this study. For the control cows, indications for HT such as disproportionate heel height and unbalanced sole were not corrected.

### Data Collection

The cows were assessed for locomotion scores, hock condition, BCS, and leg hygiene every month for 9 months study period. Lameness was defined as the manifestation of two successive locomotion scores of 3 or the first score of 4 or 5 (30). Cows fulfilling the lameness definition were immediately (on the day of lameness diagnosis) examined in the HT facility for hoof health. Thereafter, the lame cows were treated according to the farms' management protocol, which entailed a therapeutic trim, administration of a non-steroidal and anti-inflammatory drug, placement of hoof block on the healthy claw, and local antibiotic agent depending on the lesion type. The ICAR claw health atlas (31) was used as a guide for the lesion diagnosis. Lesions such as sole ulcer, sole hemorrhage, double sole, white line disease, toe ulcers, and thin sole were categorized as non-infectious lesions, whereas infectious lesions included digital dermatitis, interdigital dermatitis, heel horn erosion, and swollen coronet. Lesions such as corkscrew claw, interdigital hyperplasia, and wall fissures were categorized as “others.” All the non-lame cows that remained in the study were examined for hoof lesions after the follow-up period.

### Data Analysis

All statistical analyses were conducted using the IBM SPSS version 24.0 (Armonk, NY, USA: IBM Corp.). Descriptive statistics were used to simplify the characteristics of the enrolled cows. Mean and standard deviations were used to summarize the continuous variables, whereas categorical variables were presented in median, interquartile range, and percentages. The cows with complete locomotion scores and other animal-based measures either before censoring or throughout the study period were included in the final analysis. Those with missing data or culled before censoring or the end of the study period were not included. The outcomes (lameness incidence and lesion prevalence) were determined at cow levels using descriptive statistics. The incidence rate of lameness was calculated as the number of new cases divided by the total number of cows at risk multiplied by the time at risk. Lesion prevalence was calculated based on the total number of cows affected with either one or more hoof lesions to the total number of observed cows at the end of the study.

Survival analysis was used to evaluate lameness incidence in the trimmed and control groups. The time of lameness diagnosis was the date of the second successive lame locomotion score (LS = 3) or the first severe lameness score (LS = 4). The difference in time to lameness (measured in months) between the study groups was evaluated using Kaplan-Meier analysis. Univariable Cox proportional regression models were first constructed to evaluate the relationship between lameness incidence and the covariates: parity (first, second and greater parity), breed, DIM (≥200 DIM and within 30 DIM during enrollment), HCS (normal, hair loss, and swelling/ulcer), leg hygiene (clean, dirty and very dirty) and BCS (≤2.5, 2.6–3.4, and ≥3.5) and hoof overgrowth (present or absent). For the four latter covariates, records used in the regression analysis were the respective scores or observations either at the point of censoring (for lame cows) or at the end of the study period (for non-lame cows). Farms were introduced in the model as random effects. In the next stage, covariates were introduced into the multivariable cox proportional regression model if the *P*-value was <0.10. A forward conditional method was applied and changes in the remaining coefficients were checked as factors were added into the model. *P*-value <0.05 was used for the final model. Risk estimates were presented as hazard ratios with a 95% confidence interval. Interaction between farm groups and other predictors was checked. The proportional hazard function in the final model was assessed based on the Schoenfeld residuals test (32).

Hoof lesion prevalence was analyzed by including all the cows diagnosed with lameness during and at the end of the study period. The outcome was the odds for any hoof lesion either during or at the end of the follow-up period. Due to the low prevalence of infectious hoof lesions (<10%), analysis was only conducted for non-infectious types. Therefore, cows having a non-infectious hoof lesion and those without lesions were included in the regression models. Binary logistic regression models were conducted for all the enrolled cows. A similar two-step model building process described earlier was used to construct the final multivariable logistic regression models. Farms were considered as a random effect in the final model, whereas groups (trimmed vs. control) were treated as a fixed effect. Biologically plausible interactions were checked in the main effects, however, none was retained (all predictors had *P* > 0.05). The final model fit was selected based on the lowest Akaike's information criterion.

## Results

### Descriptive Analysis

A total of 520 cows were enrolled in the study (HGR = 170, CON-GR = 97; IS; HNGR = 124, CON-NGR = 129). However, 44 cows were either culled or had missing data during the study and were not included in the final analysis. Descriptive statistics of the cows (*n* = 476) with complete data are presented in Table 2. The total number of enrolled cows in NGR herds was 232 with 72.8 and 27.2% in trimmed (HNGR) and control (CON-NGR), respectively. In GR herds, the proportion of cows in HGR and CON-GR were 65.6% (*n* = 160) and 34.4% (*n* = 84), respectively. The majority of the cows in both groups were Australian Friesian Sahiwal breed (GR vs. HGR; 69.3 vs. 84.4%), and equal proportions (75.8%) had BCS between 2.6 and 3.4 during enrollment. Also, both groups had similar proportions of cows with normal hock conditions, clean legs, and overgrown hooves. Overall, the major indication for HT was overgrown dorsal wall length (51.7%).

**Table 2:** Characteristics of all the enrolled cows (*n* = 520) in grazing and non-grazing dairy farms.

	**Non-grazing farms**				**Grazing farms**			
**Factors**	**Farm A**	**Farm B**	**Farm E**	**Total (%)**	**Farm C**	**Farm D**	**Total (%)**	**Overall (%)**
	**Freq**.	**Freq**.	**Freq**.		**Freq**.	**Freq**.		
Breed								
Friesian Sahiwal	52	75	69	196 (84.4)	79	90	169 (69.3)	365 (76.6)
Jersey Friesian	36	–	–	36 (15.6)	33	42	75 (30.7)	111 (23.4)
Parity								
1	40	61	18	119 (51.2)	29	37	66 (27.0)	185 (38.8)
2	40	14	43	97 (41.8)	60	63	123 (50.4)	220 (46.2)
≥3	8	0	8	16 (7.0)	23	32	55 (22.6)	71 (15.0)
BCS								
2.5–2.9	7	7	0	14 (6.0)	4	2	6 (2.4)	20 (4.2)
3.0–3.4	66	63	47	176 (75.8)	80	105	185 (75.8)	361 (75.8)
3.5–4.0	15	5	22	42 (18.1)	28	25	53 (21.7)	95 (19.9)
Hock condition								
Normal	68	65	56	189 (81.5)	104	110	214 (87.7)	403 (84.6)
Hair loss	19	10	12	41 (17.7)	8	21	29 (11.8)	70 (14.7)
Swelling/ulcer	1		1	2 (0.8)	0	1	1 (0.4)	3 (0.7)
Leg hygiene								
Normal	50	36	58	144 (62.1)	89	78	167 (64.3)	311 (65.3)
Dirty	24	35	11	70 (30.2)	22	40	62 (21.3)	132 (27.7)
Very dirty	14	4		18 (7.7)	1	14	15 (61.5)	33 (6.9)
Hoof overgrowth								
Absent	36	52	33	121 (52.2)	86	63	149 (61.6)	270 (56.7)
Present	52	23	36	111 (47.8)	26	69	95 (38.9)	206 (43.3)
Group								
Trimmed	45	35	38	118 (72.8)	89	71	160 (65.6)	278 (58.4)
Control	43	40	31	114 (27.2)	43	41	84 (34.4)	198 (21.6)
Trimmed foot (per cow)								
All	28	38	11	77 (65.3)	24	73	97 (60.1)	174 (36.5)
Both rear	9	–	24	33 (27.9)	46	9	55 (34.4)	88 (18.4)
Both rear and one front foot	8	–	–	8 (6.8)	–	8	8 (5.0)	16 (3.4)
Indication for HT								
Dorsal wall length	28	35	17	80 (67.7)	23	41	64 (40.0)	144 (51.7)
Heel height	4	2	1	7 (5.6)	22	37	59 (36.9)	66 (23.7)
Unbalanced sole	13	1	17	31 (26.3)	25	12	37 (23.1)	68 (24.4)
Days in Milk (Mean ± SD)	92.0 ± 102.7	130.6 ± 115.3	148.2 ± 111.7	109.1 ± 111.4	117.8 ± 118.8	115.8 ± 113.4	126.5 ± 115.3	

*Freq., frequency; SD, standard deviation; HT, hoof trimming; BCS, body condition score*.

*Indications for HT and trimmed foot per cow were only presented for the trimmed cows in grazing and non-grazing farms*.

### Lameness Analysis

**Figure 1:**
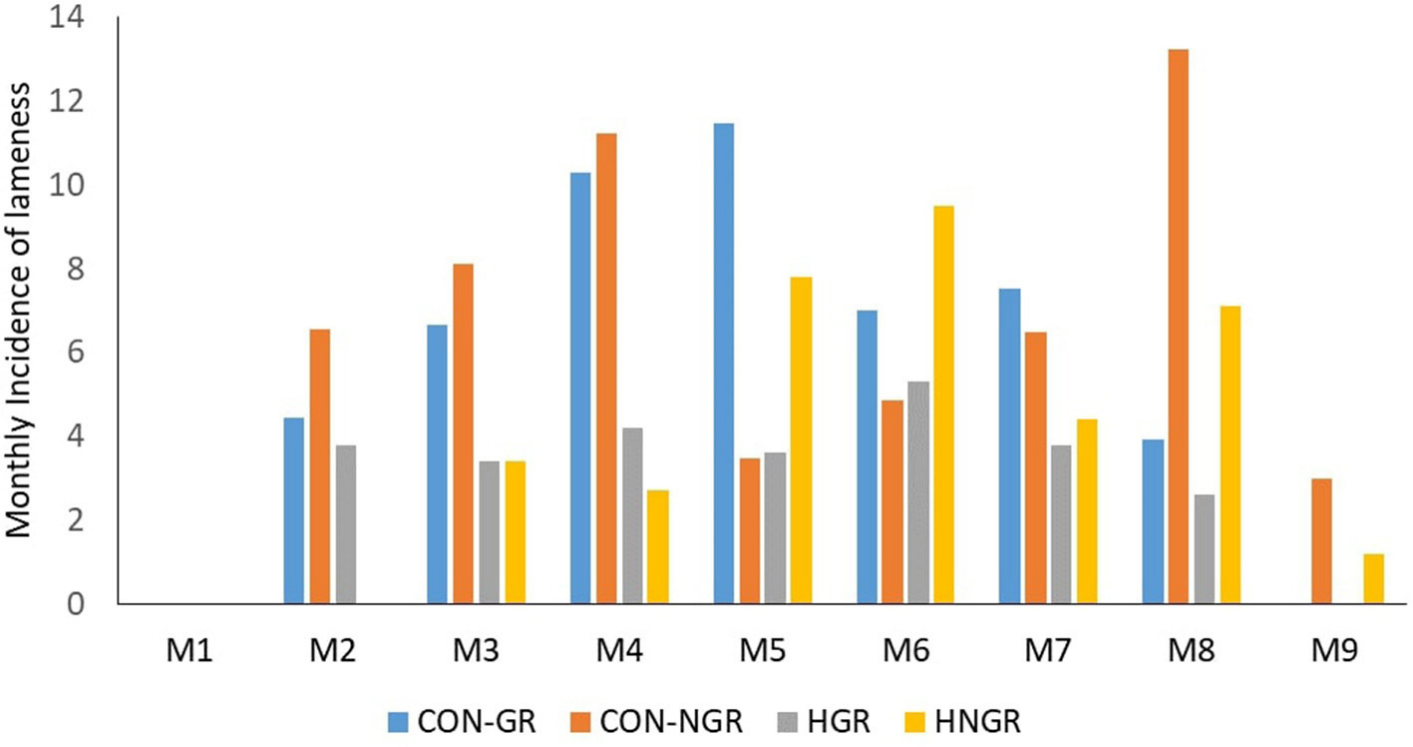
Incidence rate of lameness in hoof-trimmed and control cows in non-grazing and grazing herds during the 9 month study period (M1 = first month post-enrollment, M9 = ninth month post-enrollment).

**Figure 2:**
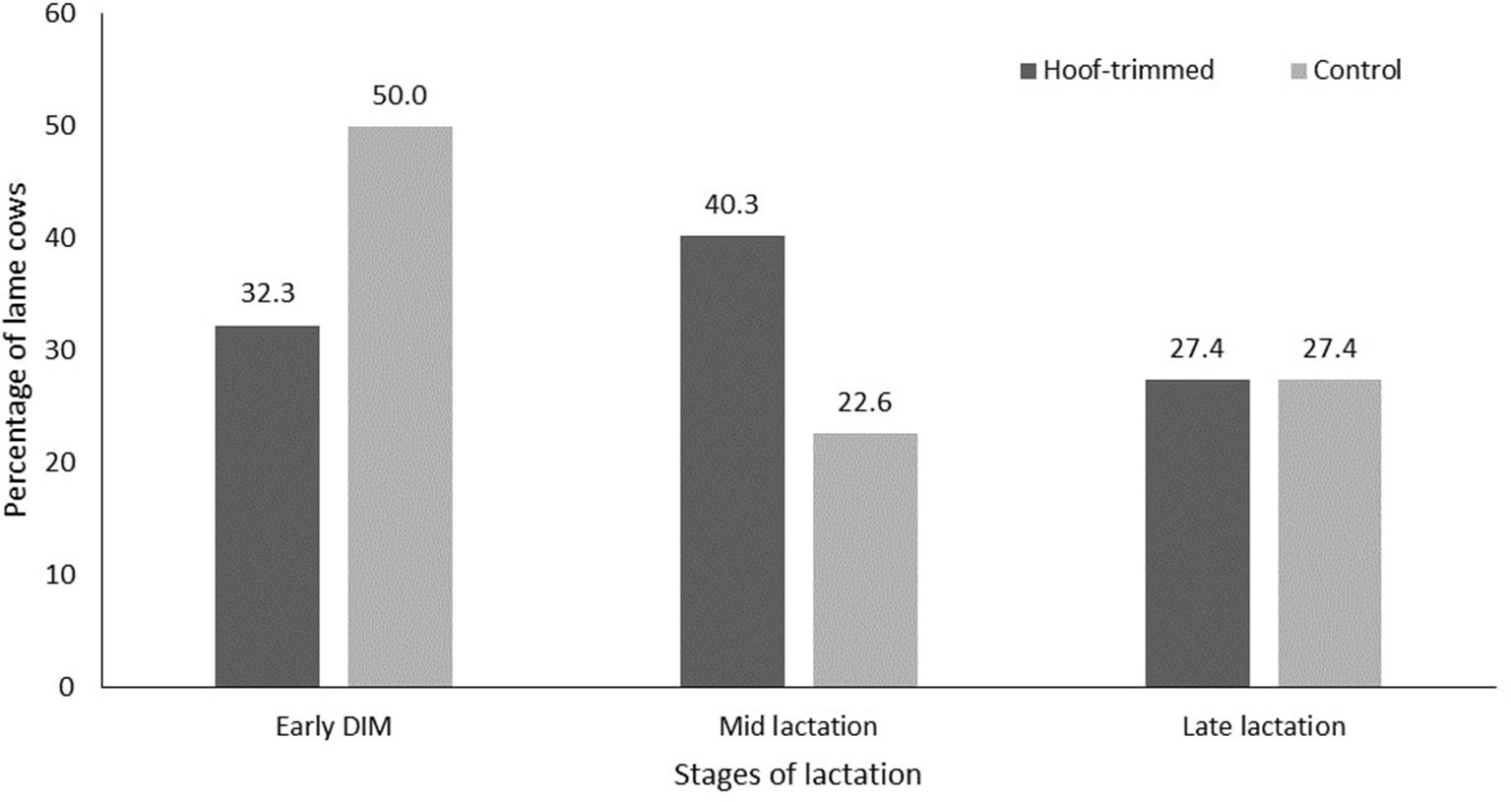
Percentage of lame cows among the hoof-trimmed and control cows at various stages of lactation during the study.

The monthly incidence rate of lameness in HGR and HNGR ranged from 5 to 9% throughout the study period; however, CON-GR and CON-NGR had higher monthly lameness incidence rates (12 vs. 13%) in the fourth and 7th month into the study (Figure 1). Overall, the incidence rate of lameness in the enrolled cows all through the study period was 36.3 cases/100 cows/per month. The corresponding rate of lameness in HGR, HNGR, CON-GR, and CON-NGR were 27.4, 31.9, 48.4, and 45.8 cases/100 cows/per month, respectively (Table 3). Time to first lameness event measured in months was significantly higher in HGR (mean ± S.E; 8.12 ± 0.15, *P* = 0.04) compared to CON-GR (7.36 ± 0.26), and in HNGR (8.05 ± 0.16, *P* = 0.03) compared to CON-NGR (7.39 ± 0.23). The majority of the lameness events in trimmed and control cows were observed during early lactation (50%) and mid-lactation (40.3%), respectively (Figure 2).

**Table 3:** Time to first lameness event and lameness incidence rate in trimmed and control cows from grazing (*n* = 2) and non-grazing (*n* = 3) dairy farms.

	**Time to first lameness (months)**			**Mantel-Cox**				
	**Mean**	**S.E**	**95% CI**	**Chi-square**	***P*-value**	**Lame cows**	**ATE**	**Incidence rate**
Grazing cows								
HGR^a^	8.12	0.15	7.69–8.32	6.37	0.01	39	142.3	27.4/100
CON-GR^b^	7.36	0.26	6.86–7.87			33	68.1	48.4/100
Non-grazing cows								
HNGR^a^	8.05	0.16	7.74–8.35	3.76	0.05	34	106.5	31.9/100
CON-NGR^b^	7.39	0.23	6.95–7.83			45	98.6	45.8/100
**Overall^c^**	**7.75**	**0.09**	**7.56–7.95**	**10.61**	**0.001**	**151**	**415.5**	**36.3/100**

a,b *Groups with significant difference in time to first lameness have different superscripts*.

*ATE, animal time events; SE, standard error*.

C *Comparison between trimmed and control cows irrespective of management systems*.

*Total number of cows in trimmed cows: HGR = 160, NGR = 118*.

*Total number of cows in Control cows: CON-GR = 84, CON-NGR = 114*.

Factors in the univariable Cox regression model included BCS, hock condition, lesion presence, and farms, but hock condition was not retained in the final multivariable model. Cows with thin BCS (≤2.5) had a higher risk for lameness (Hazard ratio; HR = 3.05, 95% CI 1.24–7.46) compared to those with good BCS (Table 4). The risk of lameness was higher in cows affected with hoof lesions (either infectious, non-infectious or both) than those with a healthy hoof. Overall, lameness risk varied between farms with farm C recording higher risk compared to farm E.

**Table 4:** Final multivariable cox regression models for factors associated with time to first lameness event in 476 cows from five farms in Peninsular Malaysia.

**Factors**	**B**	**SE**	**Wald**	***P*-value**	**HR**	**95% CI**	
BCS			18.35	<0.001			
≤2.5	1.15	0.45	5.96	0.01	3.05	1.24	7.46
2.6–3.4	0.70	0.45	2.44	0.11	2.02	0.83	4.83
3.5–3.9	0.08	0.46	0.03	0.86	1.08	0.43	2.63
≥4.0					Ref		
Lesion			126.72	<0.001			
Non-infectious	3.50	0.32	117.26	<0.001	33.18	17.61	62.55
Infectious	3.66	0.37	97.60	<0.001	38.99	18.85	80.65
Both	2.99	0.48	37.74	<0.001	20.02	7.69	52.08
No lesion					Ref		
Farms			10.22	0.043			
Farm A	0.23	0.29	0.61	0.43	1.26	0.70	2.26
Farm B	0.03	0.30	0.01	0.92	1.03	0.57	1.85
Farm C	0.72	0.28	6.24	0.01	2.04	1.16	3.58
Farm D	0.19	0.29	0.44	0.50	1.22	0.67	2.19
Farm E					Ref		

*BCS, body condition score; HR, hazard ratio; SE, standard error; CI, confidence interval; Ref, reference category*.

### Hoof Lesions Analysis

Table 5 shows the prevalence of hoof lesions in all the enrolled cows (i.e., both lame and non-lame) at the end of the study. The prevalence of hoof lesions in the enrolled cows was 36.9% (176/476), with a higher prevalence in CON-GR (48.8%) than HGR (23.2%). Likewise, CON-NGR recorded a higher prevalence (52.6%) of hoof lesions compared to HNGR (32.2%). The majority of hoof lesions were non-infectious in grazing (HGR vs. CON-GR; 21.3 vs. 33.3%) and non-grazing herds (HNGR vs. CON-NGR; 25.0 vs. 40.4%). The prevalence of infectious hoof lesions in HGR and CON-GR was 1.9 and 15.4%, respectively. However, similar proportions of infectious hoof lesions were recorded in HNGR (6.8%) and CON-NGR (7.0%).

**Table 5:** Hoof lesion prevalence in trimmed and control cows from grazing (*n* = 2) and non-grazing (*n* = 3) dairy farms.

	**Frequency of hoof lesions**												
	**SU**	**SH**	**WLD**	**TU**	**TS**	**DD**	**SC**	**Others**	**Total**	**Cows**	**Prevalence (%)**	**NIF**	**IF**
Hoof-trimmed													
HGR	14	5	6	8	1	3	0	0	37	37	23.2	21.3	1.9
HNGR	17	7	10	5	1	6	2	2	50	38	32.2	25.0	6.8
Control													
CON-GR	11	5	9	4	1	10	3	4	47	41	48.8	33.3	15.4
CON-NGR	19	7	16	3	2	6	2	6	61	60	52.6	40.4	7.0
**Overall**	**61**	**24**	**41**	**20**	**5**	**25**	**7**	**12**	**195**	**176**	**36.9**		

*SU, sole ulcer; SH, sole hemorrhage; WLD, white line disease; TU, toe ulcer; TS, thin sole; DD, digital dermatitis; SC, swollen coronet; NIF, non-infectious hoof lesions; IF, infectious hoof lesions*.

*Total number of cows in trimmed group: GR = 160, NGR = 118*.

*Total number of cows in Control group: GR = 84, NGR = 114*.

*More than one lesion per cow was included*.

Table 6 shows the estimated associations between covariates and having a non-infectious hoof lesion. Factors in the multivariable model included parity, BCS, hock condition, hoof length and treatment groups. Lower parity (Odds ratio; OR = 0.41, 95% CI 0.19–0.88), normal hock condition (OR = 0.06; 95% 0.01–0.29) and absence of overgrown hoof (OR = 0.47; 95% 0.28–0.79) were protective against non-infectious hoof lesion. Factors that increased the odds of non-infectious hoof lesions included low BCS (BCS ≤ 2.5) (OR = 19.71, 95% CI 6.39–60.81) and belonging to the control group (OR = 3.25; 95% 1.92–5.53) relative to those with BCS ≥ 4.0 and trimmed cows, respectively.

**Table 6:** Univariable and multivariable logistic regression models showing the significant factors associated with non-infectious hoof lesions identified at the end of study period.

	**Univariable model**						**Multivariable model**		
**Factors**	***B***	**SE**	***P*-value**	**OR**	**95% CI**		**OR**	**95% CI**	
Parity			0.03						
1	−0.94	0.40	0.01	0.39	0.17	0.85	0.41	0.19	0.88
2	−0.95	0.38	0.01	0.38	0.18	0.82	0.40	0.19	0.84
>2				Ref			Ref		
BCS			0.001						
≤2.5	2.99	0.57	0.001	20.0	6.43	62.37	19.71	6.39	60.81
2.6-3.4	1.63	0.55	0.003	5.10	1.71	15.18	5.21	1.76	15.43
3.5-3.9	0.18	0.57	0.744	1.20	0.39	3.69	1.20	0.39	3.69
≥4.0				Ref			Ref		
Hock condition			0.005						
Normal	−2.58	0.81	0.002	0.07	0.01	0.37	0.06	0.01	0.29
Hair loss	−2.19	0.82	0.008	0.11	0.02	0.56	0.09	0.01	0.44
Ulcer/swelling				Ref			Ref		
Hoof length										
Normal	−0.73	0.26	0.006	0.48	0.28	0.81	0.47	0.28	0.79
Overgrown				Ref			Ref		
Group										
Not trimmed	1.23	0.27	<0.001	3.44	2,00	5.92	3.25	1.92	5.53
Trimmed									

*BCS, body condition score; OR, odds ratio; SE, standard error; CI, confidence interval; Ref, reference category*.

## Discussion

This study adds to the body of knowledge on the impact of the five-step Dutch trimming method on lameness incidence and lesion prevalence in grazing and non-grazing cows. Overall, the incidence rate of lameness in hoof-trimmed cows from GR and NGR was 27.4 cases/100 cows/month (3.6 cases/cow/year) and 31.9 cases/100 cows/month (4.2 cases/cow/year), respectively. The present result is consistent with the lameness incidence rate reported in cows from freestalls during dry periods (4.2 cases/cow/year) (33) but lower compared to 7.4 cases/cow/year recorded in lactating cows from a freestall herd (34) and pooled lameness incidence in grazing herds (64.6/100 cow-years) (16). The high lameness incidence rate could be attributed to the enrollment of primiparous and older cows, which are more susceptible to lameness episodes, management practice, and the presence of control groups that were not trimmed during lactation.

High milk-yielding cows are at higher risk of becoming lame (10, 23), but this was not the case in the present study. Although the enrolled cows in this study are expected to be high producers based on their genetics, the milk yield was relatively lower compared to the same breed of cows in other related studies (19, 29). Factors such as poor heat abatement strategies and nutrition may contribute to the low milk yield in the study population (9, 13). Based on the high lameness incidence rates, especially in the untrimmed cows, the finding suggests that other management factors may play a role in gait disturbance and the onset of hoof lesions on Malaysian dairies. A recent study found that floor designs around the milking pen, walkways, and resting pen, poor herd hygiene, and lack of hoof care influenced the risk of foot lesions (35). Hence, the application of HT as a management strategy may reduce the occurrence of hoof lesions such as sole ulcers and white line disease, which are the predominant causes of lameness in dairy cows in Peninsular Malaysia (35). Nevertheless, a more holistic strategy involving improved housing conditions, stall designs, and routine hoof inspection and care is required.

The time to first lameness event was significantly higher in trimmed groups compared to their respective controls in grazing and non-grazing herds. The result highlights the efficacy of the HT technique as a preventive measure for lameness in lactating dairy cows. Previous studies have investigated the impact of functional trimming on lameness prevention (19, 29, 36). Mahendran et al. (36) reported no significant difference in the odds of lameness and time to first lameness event between cows that underwent pre-calving and post-calving foot trim. In another study, cows trimmed at mid-lactation had a cumulative incidence of lameness of 18% compared to control groups (24%) during late lactation (19). Findings from the reviewed studies could not be solely attributed to HT, since the studied population either had hoof lesions or were not observed for lesions presence before enrollment. Although the authors used an adaption of the functional trimming, two studies reported that preventive trimming reduced the risk of lameness during lactation in freestall and pasture-based farms (20, 37). Daros et al. (33) did not state the HT technique used in their study, but primiparous cows trimmed before enrollment had lower odds of lameness during lactation. These findings are consistent with our results when comparing the incidence rate of lameness between trimmed and control cows. However, our result gives more insight on the impact of HT, since all cows in the present study were evaluated for healthy hooves and sound locomotion before enrollment. Application of functional trimming in the present study might have assisted to better the cows' gait due to improvement in weight distribution (38), frictional properties at the floor-claw interface, and preserving hoof dimensions after achieving the proper hoof length and sole thickness (17, 39).

The majority of lameness events in trimmed and control cows were observed during mid-lactation and early lactation (within 120 DIM), respectively. The reason for this finding is not fully understood. Nevertheless, early lactation is identified as a high-risk period for lameness due to factors such as negative energy balance, peri-calving hormonal changes affecting the hoof horn tissues, and challenges associated with peak lactation (40, 41). This might explain the higher lameness events in control cows at early lactation, whereas lameness episodes in trimmed cows might be delayed until mid-lactation due to the protective effect of HT.

Lesion prevalence at the end of the study period was lower in HGR (23.2%) compared to CON-GR (48.8%), as well as in HNGR (32.2%) compared to CON-NGR (52.6%). This result reflects the potential benefits of HT in grazing and non-grazing cows. For instance, the incidence of hoof lesions was lower in herds where trimmed cows spent more time on pasture (42). Pasture access as short as 4 weeks was associated with a higher tendency to bear weight on the affected claw, improved tracking up, and improved gait score of lame cows (43). In addition, HT enhances even weight distribution between the medial and lateral claws and restores proper sole thickness (17, 38, 39). These events might explain the delay in the onset and lower prevalence of hoof lesions in trimmed compared to non-trimmed cows in the present study.

Specifically, the majority of the lesions were non-infectious (i.e., hoof horn lesions) which corroborates the results from other related studies conducted in Malaysia (44, 45). Another indication of the benefits of HT in delaying the onset of hoof horn lesions was the lower prevalence in trimmed cows compared to controls under both management systems. This result is consistent with the findings of Manske et al. (20) and Gomez et al. (37), where an adaptation to functional trimming was protective against hoof horn lesions. Cows trimmed around drying off were found to have lower odds of sole ulcers (20% lower) in the subsequent lactation (21). The final step in the functional HT is the formation of a hollow dish (i.e., increased paring) around the solar area adjacent to the axial aspect of the hoof, which is regarded as the typical sole ulcer site (15, 17). Although we did not assess the longitudinal changes of sole thickness, the last step in the HT procedure might have reduced the pressure directed unto the corium during the risk period for hoof horn lesions (17).

In contrast, the prevalence of infectious hoof lesions was low in all the trimmed and control groups. Previous studies have highlighted low prevalences of digital dermatitis, heel horn erosion, and foot rot in Malaysian dairy farms (44, 45), which could play a role in the present findings. Besides, all the enrolled farms used footbaths as a lameness control practice. However, the HT may also contribute to the finding, as such intervention was reported to reduce the occurrence of digital dermatitis in pasture-based herds (46), while trimmed cows provided with pasture access had a lower prevalence of infectious foot lesions (43). The HT technique might have restored a proper heel height; thus, reducing the exposure to slurry, interdigital irritation, and subsequent lower odds of infectious lesions. A more controlled study is required to elucidate such speculation since the present study enrolled multiple farms with varying levels of herd hygiene. Furthermore, the fact that both trimmed and untrimmed cows had low prevalences of infectious hoof lesions limits our understanding of the effectiveness of the HT procedure.

The risk of lameness was higher in underconditioned cows and those affected with hoof lesions, with the incidence varying between farms. Previous researches have consistently demonstrated that low BCS predisposes cows to lameness and the other way around (47, 48). In addition, loss of BCS and increase of BCS at calving could influence the risk of future lameness events and the chance of recovery from lameness (49). BCS loss promotes thinness of the digital cushion and instability of the pedal bone; thus, supporting the pathogenesis of hoof horn lesions causing lameness (11, 12). These events are further augmented by the absence of HT, improper trimming, and long intervals between trimmings (50). Good BCS was a criterion for enrollment of cows in this study, however, the observation points (every month) limit our knowledge on the event direction. Cows could have been underconditioned prior to lameness onset or the other way around.

Lameness risk varied between farms in this study. Farm C had a higher risk of lameness compared to Farm E, but the risk was not different on other farms. The reasons for this finding are not clearly understood since important animal-based factors such as BCS, DIM, hock condition that could influence the risk of lameness were considered during enrollment. Nevertheless, a higher number of cows on farm C received HT on both hind hooves compared to other farms. Hoof traits such as overgrown dorsal wall length, uneven sole thickness, and disproportionate heel height may affect weight distribution between the medial and lateral claws, and heighten the risk of future lameness event (11, 17). A previous study reported a significant increase in LS in cows from few days to 2 weeks after preventive HT (51). However, the cows in the present study were observed at monthly intervals, which suggests that aside from HT, herd-level factors beyond the scope of this work might have contributed to the onset of high locomotion scores on farm C.

Lower parity was a protective factor against hoof horn lesions in the present study. Chronic degeneration of body structures such as ligaments, bone, and digital cushion are mainly associated with increasing age (52, 53). However, such changes are not common in first parity cows probably due to fewer lactations and less exposure to high-risk periods of lameness (24). Other factors that may play a role in higher risks of hoof horn lesions in older cows include relapse of such lesions in subsequent lactation (53), the onset of exostosis on the caudal aspect of the distal phalanx, and reduced protective capacity of the digital cushion following replacement by connective tissue (54, 55). For instance, previous lameness episodes were not considered as criteria during cows' enrollment in the present study. These factors may contribute to the increased odds of hoof horn lesions observed in underconditioned cows.

Cows with normal hock conditions had a lower prevalence of non-infectious hoof lesions compared to those with hock injuries. The result highlights the importance of cow comfort in lameness management. Lame cows affected with either sole ulcers or white line disease may lay down for longer periods, which may promote the onset of hock injuries especially when lying surfaces are abrasive (56). Moreover, environmental and housing factors relating to poor stall designs may influence the concurrent onset of hock injuries and hoof lesions (57, 58). A longitudinal assessment of cows with normal hock conditions and at shorter time intervals is more appropriate to ascertain the event's direction.

We acknowledge the strengths and limitations of the present study. The enrolled cows were randomly selected and placed into treatments and control groups in each farm, assessed for hoof health status before enrollment, and detailed explanation and standardization of HT technique. All the assessments were conducted by a single veterinarian. HT was conducted by the researcher in 4 of the dairy farms, whereas both professional hoof trimmers and the researcher carried out the procedure in farm D. The hoof trimmers in farm D also apply the Dutch Five-Step HT method for lameness management. Nevertheless, a hoof trimming training session involving the researcher and professional hoof trimmers (*n* = 3) was conducted before the onset of the study. The training entailed the steps and landmarks of the HT procedure and to have a standardized format. No separate training was conducted for locomotion scoring; however, a high level of agreement (Kappa coefficient = 0.80) was observed between the researcher's estimate of gait scores and available records conducted by the hoof trimmers on the same cows. These factors assisted in reducing issues related to low inter-rater reliability, confounding factors, and increased the chances of attributing changes in gait and hoof health to HT.

Nevertheless, aside from the management systems, some of the on-farm routine practices might influence lameness events during lactation. The assessment of previous lameness history in the study population was based on farm health records, which is insufficient to ascertain the true scenario before the study. Regarding the environmental conditions, data on the climatic conditions such as temperature and humidity were not collected. Although the climatic conditions in the various study areas are not expected to vary widely since they are within Peninsular Malaysia, they may impact differently on the outcomes. Furthermore, our results could not elucidate the influence of timing and frequency of preventive HT in dairy cows. Future studies might focus on these areas to improve our knowledge on the impact of HT as a preventive strategy for lameness management on dairies.

## Conclusion

This study revealed that early and late-lactation functional HT contributed to lower lameness incidence, higher survival time to first lameness event, and lower prevalence of hoof lesions compared to non-trimmed cows from grazing and non-grazing farms. Factors that also increased the risk of lameness during lactation were low BCS, presence of hoof lesion, and there was variation in lameness risk between farms. The predominant causes of lameness were non-infectious hoof lesions. Aside absence of trimming, the odds of non-infectious hoof horn lesions were higher in older and underconditioned cows, as well as those with hock injuries. Functional HT is beneficial as a lameness preventive strategy when conducted at late lactation; however, parity, body condition, and hock condition are equally important.

## Data Availability Statement

The original contributions presented in the study are included in the article/Supplementary Material, further inquiries can be directed to the corresponding author/s.

## Ethics Statement

The animal study was reviewed and approved by Institution of Animal Care and Use Committee, Universiti Putra Malaysia (Ref: UPM/IACUC/ AUP-R010/2019). Written informed consent was obtained from the owners for the participation of their animals in this study.

## Author Contributions

SR, WS, and MS contributed to conception of the work and funding. SR and MS made substantial contributions to data acquisition, analysis, interpretation, drafted and revised the work, and wrote the final version. SS-H and RM made substantial contributions to revision of the drafted manuscript. All authors approved the final version of the paper and agree to be accountable for all aspects of the work.

## Conflict of Interest

The authors declare that the research was conducted in the absence of any commercial or financial relationships that could be construed as a potential conflict of interest.
