# Supplementary material for: Preventive Hoof Trimming and Animal-Based Welfare Measures Influence the Time to First Lameness Event and Hoof Lesion Prevalence in Dairy Cows
Source: Front Vet Sci. 2021 Jun 11;8:631844. doi: 10.3389/fvets.2021.631844 (PMC8225956; doi:10.3389/fvets.2021.631844)
Supplement: Supplementary file 1 [file Table_1.DOCX]

**Supplemental Table 1**

**Items considered under explanatory factors, definition, categories and methods applied for data collection in the enrolled farms**

| Cow and herd-level factors | Definition and assessment method | Categories | Methods |
| --- | --- | --- | --- |
| Management system | Either completely confined or provision of pasture access for specific period during the day or lactation season | 2; Grazing, non-grazing | OA |
| Pasture access | Number of hours/day for external pasture access or grazing | 2; 1-3 hrs, >3hrs | OA, Interview |
| Stocking rate | Number of useable stalls to number of milking cows (Charlton et al., 2014) | 2; Normal, overstocked | OA |
| Floor type | Based on the finishing surface design in direct contact with cows’ hoof at resting area and walkways | 3; Rubber mats, concrete, others | OA |
| Floor slipperiness | Proportion of cows that slips during movement from milking to resting pen or during routine herding (Grandin, 2008; Solano et al., 2015) | 2; Non-slippery, Slippery | OA |
| Floor cleanliness | Height of manure contamination of the heel area of gum boots after walking through the feed bunk before and after scraping/cleaning (Solano et al., 2015) | 2; Clean, dirty | OA |
| Cleaning frequency | Number of times the floor areas are cleaned or scraped | 2; twice/day, >twice/day | OA |
| Scraping method | Either the usage of manual method (pumping pipes) or automated scraper | 2; manual, automated | OA |
| Exercise | An outdoor, unroofed, soil-floored, enclosed area with access to the dairy barn. | 2; Yes, No | OA |
| Hoof trimming | Practice of routine hoof trimming for prophylaxis either by veterinarian, hoof trimmer or farm staff | 2; Yes, No | Interview |
| Footbath usage | Provision of footbath area for routine disinfection of cows’ foot | 2; Yes, No | OA |
| Source of replacement cows | Source of obtaining or purchasing new animals to be introduced into the herd. | 2; Single, multiple | Interview |
| Distance to milking point | Distance covered by cows when moving from resting barns to milking parlor, measured in meters. | Not categorized | OA |
| Milking frequency | Number of times the cows are milked per day | 2; twice/day; >twice/day | Interview |
| Season | Period in which a particular farm was visited | 2; dry and wet |  |
| Parity | Number of calvings | 3; first, second, above second parities | FR |
| Breed | Dairy cattle breeds | 3; Australian Friesian Sahiwal (AFS), Jersey Friesian | FR,CA |
| Leg cleanliness | Based on the degree of manure contamination around the upper and lower limbs and upper flank region (Vasseur et al., 2015) | 3; clean, dirty, very dirty | CA |
| Hock condition | Based on the condition of the area around the tarsal (hock) and carpal (knee) joints (Gibbons et al., 2012) | 3; healthy, hair loss, ulcerated | CA |

OA = On-farm assessment, CA = cow assessment, FR = farm records
